# Endoscopic ultrasound-guided removal of a toothpick from outside the gastrointestinal tract

**DOI:** 10.1055/a-2587-8656

**Published:** 2025-04-29

**Authors:** Guoyao Sun, Zhuo Yang, Wen Jia, Haoran Li

**Affiliations:** 1 659860 Department of Endoscopy, General Hospital of Northern Theater Command, Shenyang, China

A 60-year-old woman presented to our hospital with a one-month history of abdominal pain. She was afebrile and without systemic features of infection (white cell count 4.1 × 0^9 cells/L and C-reactive protein 1.74 mg/L). Abdominal computed tomography (CT) revealed a linear, slightly hyperdense structure in the gastric antrum region extending outside the gastric lumen ( Fig. 1 ). The patient denied swallowing any foreign objects. A submucosal eminence was observed on the posterior wall of the gastric antrum ( Fig. 2 **a** ). We performed endoscopic ultrasound for localization ( Fig. 2 **b** ) and marked it with a clip.

**Fig. 1:**
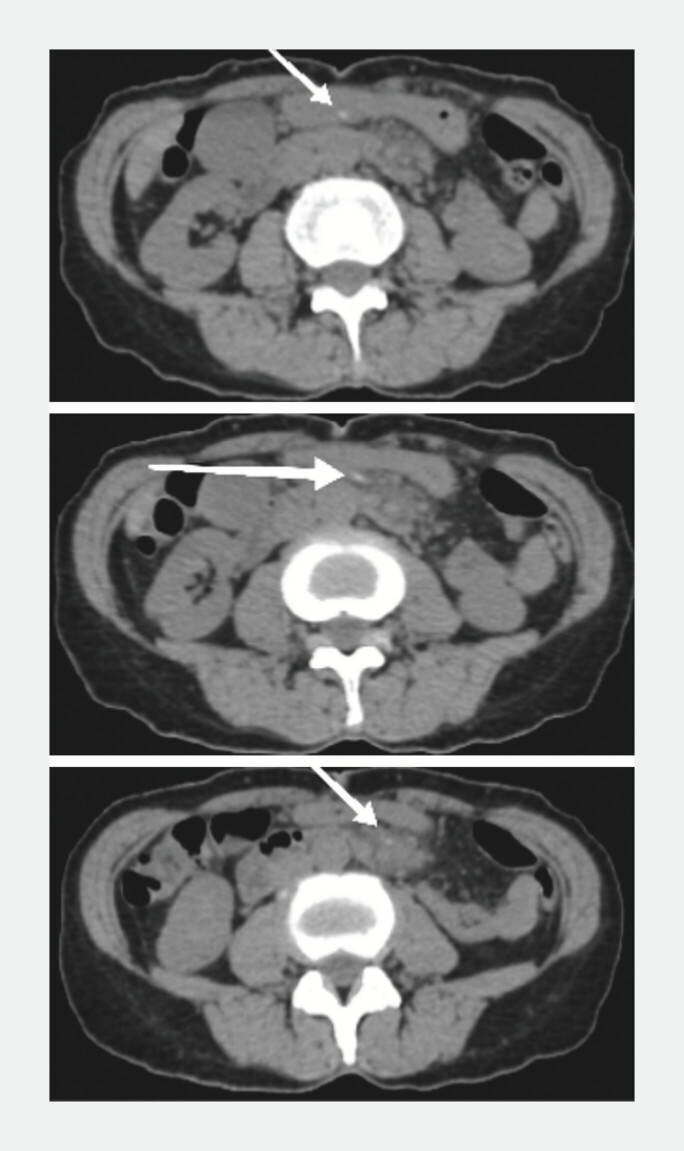
Abdominal computed tomography (CT) revealed a linear, slightly hyperdense structure in the gastric antrum region, extending outside the gastric lumen.

**Fig. 2:**
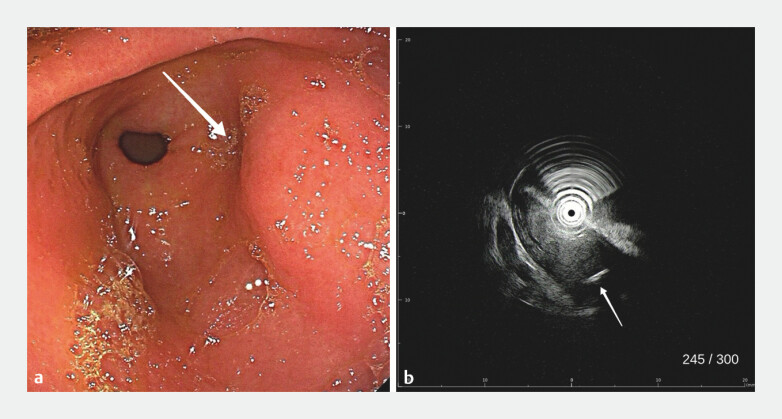
**a** A submucosal eminence was observed on the posterior wall of the gastric antrum. **b** Endoscopic ultrasound revealed a linear, slightly hyperechoic structure with its tip located in the muscularis propria.

Using the Multi-Functional Knife (EK-416D; Anrei&Sinolinks, Changzhou, China), a submucosal dissection was performed to allow thorough exploration. After exposing the muscularis propria, no foreign body was identified. A metal clip with a rubber band was used to retract the gastric wall, providing a clear view of the surgical field. Relocalization using endoscopic ultrasound guided a precise incision, followed by an endoscopic full-thickness resection (EFTR) to explore the gastric wall. We identified a toothpick vertically penetrating the muscularis propria, which was secured and removed using biopsy forceps ( Fig. 3 ). The wound was immediately closed with several metal clips ( Video 1 ). The patient received postoperative treatment with omeprazole and cefoperazone/sulbactam and was discharged on the fourth postoperative day.

**Fig. 3:**
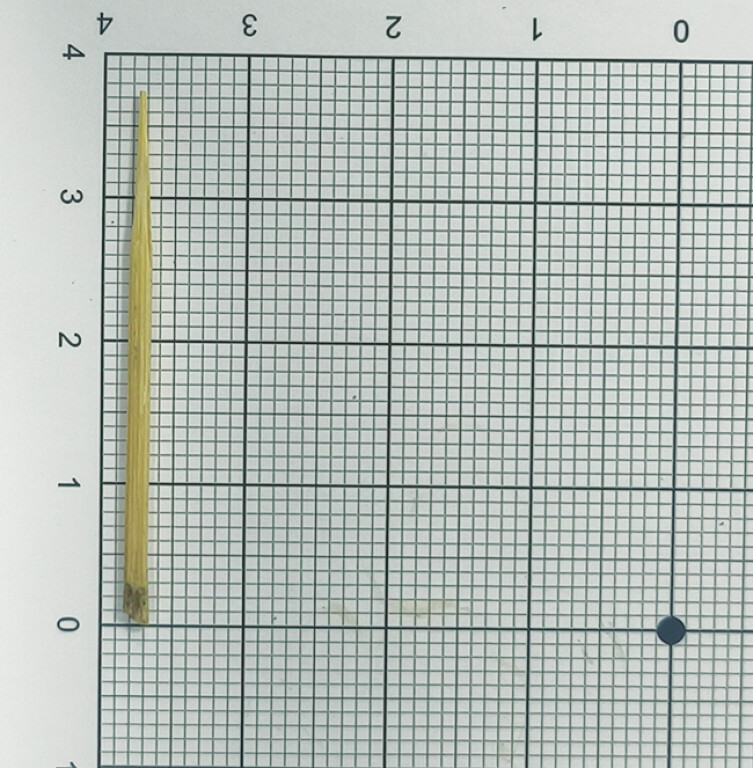
A toothpick measuring approximately 3.8 cm in length.

Endoscopic ultrasound-guided removal of a toothpick penetrating the gastric wall in a 60-year-old woman presenting with abdominal pain.Video 1

Most patients with foreign body ingestion are asymptomatic; however, some may present with endoscopic findings such as erosion, bleeding, or ulcers 1 2 . For foreign bodies embedded in the gastric wall for an extended period, as in this case, they typically present as a submucosal eminence 3 4 . In such cases, identifying the precise location of the foreign body under endoscopy can be challenging. In this case, repeated localization with EUS guided the direction of incision, enabling the successful removal of the foreign body that had penetrated beyond the digestive tract. The combined use of various minimally invasive techniques, including endoscopic ultrasound guidance, localization, traction, and EFTR, provides a safe and effective strategy for the removal of atypical foreign bodies.

Endoscopy_UCTN_Code_TTT_1AO_2AL

## Notes

Endoscopy E-Videos https://eref.thieme.de/e-videos
*E-Videos* is an open access online section of the journal *Endoscopy* , reporting on interesting cases and new techniques in gastroenterological endoscopy. All papers include a high-quality video and are published with a Creative Commons CC-BY license. Endoscopy E-Videos qualify for HINARI discounts and waivers and eligibility is automatically checked during the submission process. We grant 100% waivers to articles whose corresponding authors are based in Group A countries and 50% waivers to those who are based in Group B countries as classified by Research4Life (see: https://www.research4life.org/access/eligibility/ ). This section has its own submission website at https://mc.manuscriptcentral.com/e-videos .
